# George C Williams Prize 2015

**DOI:** 10.1093/emph/eow017

**Published:** 2016-07-04

**Authors:** Andrew F Read, Gillian R. Bentley, David Haig

**Affiliations:** ^1^Center for Infectious Disease Dynamics, Departments of Biology and Entomology, Pennsylvania State University, University Park, PA 16802, USA; ^2^Department of Anthropology and Wolfson Research Institute for Health and Wellbeing, Durham University, Durham, UK; ^3^Department of Organismic and Evolutionary Biology, Harvard University, Cambridge, MA 02138, USA

The George C Williams Prize is awarded annually by the International Society for Evolution, Medicine and Public Health to the most significant article published in Evolution, Medicine *and* Public Health. The prize is made possible by Doris Williams and contributions from other generous donors. Our committee awards the Williams Prize for 2015 to the paper *Adaptive learning can result in a failure to profit from good conditions: implications for understanding depression* by Trimmer *et al.* [1] Here we outline why.

Depression is a major and growing medical problem. It is characterized by low mood and lack of motivation among other symptoms. Depression can be caused by a pathological brain malfunction, but a variety of authors have hypothesized that some depressive behavior is adaptive, favored by natural selection in some circumstances because it increases the evolutionary fitness of the depressed person. Patients and the clinicians who treat them find it hard to accept that depression could ever be a good thing. A contrasting view is that depression needs no special evolutionary explanation because it is simply an extreme version of an adaptive behavior, a statistical aberration. Trimmer *et al.* offer a different perspective. They suggest that depression is a harmful but unavoidable side effect of innate learning rules that themselves have been favored by natural selection. Their important contribution is to suggest that understanding the learning rules can help explain why certain events sometimes trigger depression. Defining causes is the first step to finding new therapies.

The premise is that organisms need to learn when costly or risky effort will be rewarded; when it will not be rewarded, inaction is best. Mood is said to be an emotion which has evolved to regulate the amount of effort expended. When costly effort will not be rewarded, inaction—lack of motivation—makes perfect sense. But when effort will be rewarded and still the individual does nothing, he or she exhibits one important facet of depression. Trimmer *et al.* propose that this element of depression can occur as a consequence of efficiently learning about the world. Learning requires not only an assessment of current environmental conditions (are they good or bad now?) but also how often conditions change (when it is worth taking the next look at the world?). Making use of this information will on average maximize overall rewards, and so such learning will evolve. But in a subset of the population, life events can derail things. Imagine, for example, an individual who experiences good environmental conditions consistently early in life. With every bout of activity, the individual learns that environmental conditions rarely change. If that individual encounters even a short run of bad luck, s/he may assume that conditions will be bad for a long time. If the world rapidly returns to good, that individual will believe that activity is not worth it even when it is. That can generate a state of maladaptive disengagement which gets called depression and that person is now considered a patient, all because an optimal learning rule has backfired.

Trimmer *et al.*’s view that depression is a reaction of an essentially healthy and well-evolved brain to a set of definable experiences, seems to us fascinating, and is the reason we awarded their paper the G C Williams prize. We have no idea how many cases of depression their hypothesis might explain. As Trimmer *et al.* point out, it surely does not explain all cases. Serious mental disorders can involve brain abnormalities and pharmacological disturbances. Indeed, it is highly unlikely that depression has a single underlying cause. But if maladaptive learning outcomes explain a subset of cases, and those can be identified, the implications for treatment of those cases seem profound. First off, the pre-disposing causal events could occur early in life and so therapy aimed at addressing later-life events (divorce, work pressure) might be misdirected. Second, pharmacological solutions might have limited impact. Third, population-wide mental health could be enhanced if we could shape childhood experiences to minimize risk of subsequent depression.

The germ theory of disease generated immense health gains. A fruitful theory of mental disease is long overdue. Is it too much to hope Trimmer *et al.* have provided a sign post?

## References

[1] Trimmer PC Higginson AD Fawcett TW, Adaptive learning can result in a failure to profit from good conditions: implications for understanding depression. Evol Med Pub Health 2015; 2015:123–135 25916884 10.1093/emph/eov009 PMC4448095

